# Intersubject Variability of Risk from Perchlorate in Community Water Supplies

**DOI:** 10.1289/ehp.8459

**Published:** 2006-03-16

**Authors:** Doug Crawford-Brown, Bob Raucher, Megan Harrod

**Affiliations:** 1 Department of Environmental Sciences and Engineering and Carolina Environmental Program, University of North Carolina at Chapel Hill, Chapel Hill, North Carolina, USA; 2 Stratus Consulting Inc., Boulder, Colorado, USA

**Keywords:** Monte Carlo analysis, perchlorate, risk, sensitive subpopulations, water

## Abstract

This article is a brief review and summary of the estimated incremental risks (increases in hazard quotient or decreases in thyroid uptake of iodine) to pregnant women (and hence their fetuses) associated with perchlorate exposure in community water supplies (CWSs). The analysis draws on the recent health effects review published in 2005 by the National Research Council (NRC). We focus on the potential level of risk borne by the NRC-identified most sensitive subpopulation (pregnant women and hence their fetuses). Other members of the population should be at a level of risk below that calculated here, and so protection of the sensitive subpopulation would protect the general public health. The analysis examines the intersubject distribution of risks to this sensitive subpopulation at various potential drinking water concentrations of perchlorate and also draws on estimates of the national occurrence of perchlorate in U.S. CWSs to estimate the variability of risks under defined regulatory scenarios. Results suggest that maximum contaminant levels (MCLs) of up to 24.5 μg/L should pose little or no incremental risk to the large majority of individuals in the most sensitive subpopulations exposed in the United States at current levels of perchlorate in water. The protectiveness of an MCL of 24.5 μg/L depends, however, on whether the study subjects in the health effects data used here may be assumed to have been exposed to background (non-drinking water) contributions of perchlorate.

Perchlorate is an inorganic compound that has been manufactured and used as a solid rocket fuel for several decades. Initial detection of perchlorate in drinking waters was associated with proximity to military and industrial sites where the compound was produced, stored, and/or used. More recent data collection efforts suggest perchlorate is more widespread than initially thought and in some locations may be associated with sources other than military rocket fuels. In some locations, perchlorate may be present from commonly used explosive devices (e.g., fireworks, road blasting materials) and in other locations the compound may be formed naturally under suitable atmospheric and soil conditions. For example, some researchers hypothesize that lightning interactions with desert soils containing certain salt compounds may be responsible for perchlorate levels detected in western Texas (Dasgupta et al. 2005). Similar natural forces may explain the presence of perchlorate in the Atacama Desert region of Chile, and fertilizers mined from the Chilean desert may contribute to perchlorate found in some areas of the United States where those products were applied.

Perchlorate is among a class of goitergens that inhibit the uptake of iodide by the thyroid and thereby cause goiter and related iodine deficiency disorders (IDDs), including, in extreme cases, cretinism. IDD is no longer considered a public health concern in the United States because the large majority of Americans have ample iodide uptake through their normal diet to prevent IDD. There is, however, a fraction of pregnant women, between 10 and 15%, whose urinary excretion rates are elevated (Hollowell et al. 1998). If this increased urinary excretion rate is interpreted as indicating a deficit of iodine uptake (this link is not established in the cited report), these women are likely to be the sensitive subpopulation for perchlorate exposures. Iodide intake is sufficient to typically enable the thyroid to compensate and overcome any adverse effects from goitergen exposure. It is important to note that the effects of perchlorate are therefore dependent on the total pool of goitergens to which individuals are exposed.

Goitergen exposure in humans is from a variety of routes, including both water ingestion and consumption of food products found in the diet containing those with relatively high levels of nitrate (fruits, vegetables, grains, drinking water, and smoked meats), thiocyanates (broccoli, cabbage, corn, yams, sorghum, and milk), isoflavones (soy, beans, and peas), bromide (drinking water), and disulfides (onions, garlic, and peas). Goitergen intake from perchlorate exposure in water must be compared against this background of exposure to other goitergens, with risks from perchlorate resulting from the incremental effect of iodine uptake inhibition above and beyond the inhibition caused by the intake of other goitergens. Presently, the relative effectiveness of these different routes of exposure at producing decreases in iodine uptake has not been assessed, so it is not possible to specify the fraction of total decrease due solely to perchlorate exposures.

Overall goitergen exposure would need to be quite high for iodide uptake to be inhibited to a degree sufficient to elevate IDDs to a matter of health concern, although again, this level of exposure is not known at present and may be significantly lower for the sensitive subpopulation. The National Research Council (NRC) examined the risks posed by perchlorate ingestion (NRC 2005) and indicated in their executive summary, “To cause declines in thyroid hormone production that would have adverse health effects, iodide uptake would most likely have to be reduced by at least 75% for months or longer.” The mode of action for perchlorate exposure and human health risk is summarized here in the Appendix, based on the mode of action described in the NRC (2005) report.

The NRC expert panel developed an oral reference dose (RfD) of 0.0007 mg perchlorate per kilogram of body weight per day (mg/kg/day). This oral RfD is intended to reflect a safe threshold dose at which no risk of adverse health effect is anticipated for an iodide-deficient pregnant woman and any developing fetus she might be carrying. As stated by the NRC (2005): “The committee concludes that an RfD of 0.0007 mg/kg per day should protect the health of even the most sensitive populations.” This RfD is based on observing no significant inhibition of thyroid uptake of iodide at a perchlorate dose of 0.007 mg/kg/day in human subjects (Greer et al. 2002). A total uncertainty factor of 10 then was applied to ensure protection of the sensitive subpopulation: iodide-deficient pregnant women (and their fetuses). Such a sub-population could be exposed to perchlorate levels up to the RfD of 0.0007 mg/kg/day and not be expected to face a significant risk of adverse health effect. Because this is the most sensitive population, this RfD also would be protective of all other exposed individuals (including infants).

The need for a larger uncertainty factor was precluded (according to the NRC committee) by the use of a precursor to adverse effect (iodine uptake inhibition) in establishing a threshold for exposure, which was considered by the committee to represent a health-protective assumption causing the recommended RfD to be based on a no observed effect level (NOEL) rather than the more commonly used no observed adverse effects level (NOAEL). A NOAEL is by definition an adverse effect equal to or higher than a NOEL where the effect used to establish the NOEL is a precursor to the adverse effect of interest in establishing a NOAEL.

A possible argument is that a larger uncertainty factor still is warranted because we do not know the precise level at which a decrease in iodine uptake becomes adverse, and so it is possible that even small decreases may be adverse in the sense implied by the NOAEL and the lowest observed adverse effect level (LOAEL). This would be true especially in the case of women who already are iodine deficient. The authors of the present article believe this confuses the concept of an uncertainty factor as originally developed to argue for RfDs based on effects judged adverse. The question is not whether a given decrease in iodine uptake does or does not lead to adverse effects in some percentage of the population but whether such a decrease in and of itself, absent any sequellae, is to be taken as an adverse effect. Our position here is that such a decrease is not adverse in and of itself and so does not warrant the application of uncertainty factors developed originally to reason from NOAELs and LOAELs. The NRC committee appears to agree, whether explicitly or implicitly.

After the NRC report, the U.S. Environmental Protection Agency (U.S. EPA 2005) issued a statement accepting the NRC’s RfD and announcing that it had developed a drinking water equivalent level (DWEL). The DWEL converts the RfD (represented in units of mg/kg/day) into an associated concentration in drinking water (in units of micrograms per liter), taking into account the relative source contribution (RSC) from water versus other exposure routes. The DWEL was established by the EPA at 24.5 μg/L (U.S. EPA 2005) and is derived assuming a 70-kg adult consuming 2 L of drinking water per day. This gives an intake rate (of water) per unit body mass of 0.029 L/kg/day, which is slightly above the mean value for women of child-bearing age when both direct and indirect water ingestion are considered (U.S. EPA 2004, table 6.1.A2). Hence, use of this value may be considered conservative (in the sense of being health protective) for the sensitive subpopulation.

The present article places the NRC assessment into the framework of probabilistic risk assessment. The question addressed here is what the distribution of risks is in the sensitive subpopulation of pregnant women in the United States resulting from exposure to perchlorate in water from community water supplies (CWSs). The term “risk” in this article has two metrics: a hazard quotient (HQ) and a percentage reduction in iodide uptake. These risks then are examined using Monte Carlo analysis to produce intersubject variability distributions under a variety of scenarios of regulatory interest.

## Materials and Methods

### Exposure assessment

The occurrence of perchlorate in drinking waters has recently been reported in a study sponsored by the American Water Works Association’s Water Industry Technical Action Fund (Brandhuber and Clark 2004). The study relied principally on data collected under the U.S. EPA unregulated contaminant monitoring rule (UCMR), supplemented with monitoring data collected by the Massachusetts Department of Environmental Protection (MDEP), by the California Department of Health Services (CalDHS), and in Arizona and Texas (Brandhuber and Clark 2004). The results (summarized in Brandhuber and Clark 2004, table 2.1) provide estimates of the percentage of CWSs exceeding a variety of proposed maximum contaminant levels (MCLs) for perchlorate. For the U.S. EPA sampling, the percentages of CWSs exceeding 2, 4, 6, 10, and 20 μg/L were 4.1, 2.6, 1.6, 0.9, and 0.2%, respectively. For the CalDHS sampling, the percentages of CWSs exceeding 2, 4, 6, 10, and 20 μg/L were 10.5, 5.8, 3.2, 1.5, and 0.3%, respectively. For the MDEP sampling, the percentages of CWSs exceeding 2, 4, 6, 10, and 20 μg/L were 1.1, 0.8, 0.6, 0.3, and 0.0%, respectively. Data from Arizona and Texas are not included here because they did not identify whether a water source was potable or nonpotable or whether it was part of a water system. Unfortunately, the data are insufficient at present to develop a fully probabilistic population-weighted distribution of concentrations in CWSs, and so the present analysis assumes no correlation between system size (and hence size of population served) and perchlorate concentration.

The UCMR used analytic methods with a detection limit of 4 μg/L (micrograms per liter are essentially the same as parts per billion), and drew four quarterly samples from each entry point to the distribution system (EPDS) for every CWS > 10,000 persons served in the United States. Data also were collected for a sample of 771 smaller systems, but this sample may be too small to provide a sound basis for statistical inference. These data suggest a slightly higher concentration in the smallest water supplies, and so the analysis of Brandhuber and Clark (2004) may underestimate exposures (by up to 20%) in the small percentage of the population using these small systems serving fewer than 10,000 people. The results reported by Brandhuber and Clark (2004) and used here reflect the UCMR database compiled as of August 2004, when the database did not yet contain all the data from all quarters for all EPDSs. Hence, the final UCMR data set may suggest results that differ slightly from those discussed here.

The UCMR data reveal detectable amounts (≥ 4 μg/L) in 1.9% of the samples taken. Because most CWSs have more than one EPDS, and samples were taken for each EPDS, a higher percentage of CWSs (> 1.9%) were found to have at least one EPDS with detectable levels of perchlorate. The UCMR data suggest that perchlorate occurs in detectable amounts in at least one EPDS associated with 5.4% of CWSs. In systems serving > 10,000 people, perchlorate was detected in 6.1% of groundwater-based CWSs and in 4.9% of the surface-water–fed systems.

Although > 5% of large CWSs in the UCMR database had some detectable perchlorate in at least one of the EPDS-finished waters, the levels observed were generally quite low. More than two-thirds (68%) of the measurable perchlorate concentrations were in the 4–8 ppb range, and 86% were < 12 μg/L. Only 2.6% of the detected samples had concentrations > 24 μg/L (Brandhuber and Clark 2004), which is near the U.S. EPA-designated DWE of 24.5 μg/L (U.S. EPA 2005). The highest observed level in the UCMR data was 420 μg/L.

In Massachusetts, samples were analyzed with a more sensitive detection limit that yielded quantifiable results ≥ 1 μg/L and “trace” observations for levels < 1 μg/L. This method revealed that 2.4% of treated drinking water samples contained detectable levels of perchlorate. However, the vast majority of the Massachusetts detections in treated waters were at or near the 1-μg/L limit of detection: 66% of detects in treated drinking water were at trace levels (≤ 1 μg/L), 83% of detects were ≤ 2 μg/L, and 90% were ≤ 4 μg/L (Brandhuber and Clark 2004).

The above data were fit by a lognormal distribution. The resulting distribution is characterized by a median of 0.03 μg/L and a geometric standard deviation (GSD) of 13. The assumption here is that the properties of the distribution identified at the higher levels of exposure (≥ 1 μg/L) continue to apply in water supplies at concentrations below the detection limit.

Based on the NRC review, potential for risk arises only if a person from the sensitive subpopulation ingests perchlorate at an incremental rate (i.e., above background) that exceeds the identified threshold for effect. The average daily rate of intake (ADRI) for any individual is based on how much tap water they consume, the concentration of perchlorate in their tap water, and their body weight. These three factors vary across the U.S. population of pregnant women. Using available data, the distributions for these variables can be included in a Monte Carlo analysis to develop a combined distribution of ADRI values across this subpopulation. The distribution of water ingestion rates used here is based on total CWS consumption values for adults established by the U.S. EPA (2004), which provides values associated with given percentiles of the variability distribution.

Data on water ingestion for pregnant women were too limited to use reliably in this analysis, but the existing data suggest that using the data for U.S. adults does not understate exposures in pregnant women. As demonstrated in the U.S EPA *Exposure Factors Handbook* (U.S. EPA 1997), the difference in intake rates of tap water for the general population of women of child-bearing age and pregnant women is small (mean of 1.16 vs. 1.19 L/day), and so the former is assumed to approximate the latter intake rates in this analysis. The distribution of body weight for 25-year-old women (representing women 18–40 years of age, who largely make up the child-bearing–age population) is taken from the U.S. EPA *Exposure Factors Handbook* (U.S. EPA 1999). The data on water ingestion rate per unit body weight described above then were fitted by a lognormal distribution, with a best fit showing a median of 0.0182 L/kg/day and a GSD of 1.8. This distribution is consistent with the mean value assumed in regulatory calculations.

The U.S. EPA typically employs an RSC of drinking water, expressed as the percentage of total contaminant dose that is provided by drinking water, to estimate total risk from all routes of exposure (i.e., aggregate risk). These RSCs for drinking water generally are in the range of 20–80%. The relevance of applying an RSC here depends on how one interprets the human subject perchlorate study conducted by Greer et al. (2002) that forms the basis of the risk coefficients. An RSC is appropriate when the study on which risk coefficients are based includes only exposures through one route, whereas exposures through other routes will be present in exposure situations envisioned in regulatory decisions (and so must be factored in when regulating exposures by the first route). If one assumes that the individuals in the study by Greer et al. (2002) were exposed to the same background levels of perchlorate as the rest of the U.S. population (there is nothing in their diets or in the study design to preclude this), then no further RSC adjustment is needed to reflect total exposures via all routes because the risk coefficient from the study already reflects the incremental risk from ingestion of perchlorate in water above and beyond the contributions to perchlorate exposure via the other routes. Similarly, because the study population presumably was exposed to the complement of goitergens other than perchlorate, the study by Greer et al. (2002) also reflects the incremental risk from ingestion of the goitergen perchlorate above and beyond the contributions from these other goitergens. This is the scenario we employ in our analysis. Unfortunately, adequate data are not available at present to estimate the RSC for water exposures reliably.

### Risk characterization

A standard metric of potential health risk for threshold contaminants like perchlorate is the HQ. The HQ is equal to the estimated ADRI (in units of milligrams per kilogram per day) divided by the RfD. An HQ value of 1.0 thus means that a person is receiving an ADRI equal to the RfD. Any HQ value ≥ 1.0 indicates that exposure is at or below the “no risk” threshold (the term “no risk” here meaning a risk judged to be nonsignificant), and thus no significant risk of adverse health effect is anticipated. An HQ value > 1.0 indicates an ADRI above the RfD and suggests that there may be some nonzero risk of adverse health effect (although, because of the uncertainty factors in the RfD, which produce a margin of safety, the risk may be zero even for exposures yielding HQ values < 1.0). In the present article, we use the value of RfD suggested by the NRC (2005): 0.0007 mg/kg/day.

**Figure 1:**
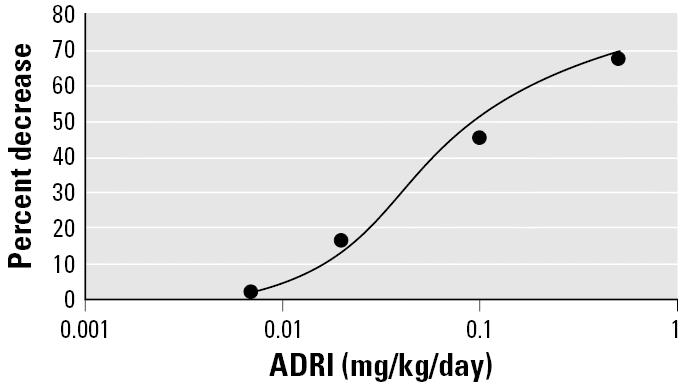
Data (circles) on decrease in iodine uptake in the thyroid versus ADRI for perchlorate in healthy males and females, averaged over the sexes (the difference between sexes is not statistically significant). Data from Greer et al. (2002). The line is the best-fitting model as discussed in the text.

Another measure of effect used in this analysis is the estimated percent decrease in iodide uptake by the thyroid (the critical effect used originally to establish the RfD). This is estimated based on fitting a dose–response curve to the data from Greer et al. (2002), relating the ADRI to the percent decrease in iodide uptake. The resulting curve is shown in Figure 1. The best model fit is as follows:


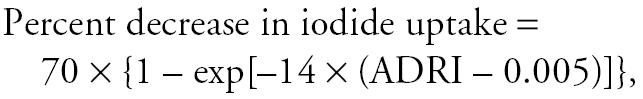


where ADRI is in units of mg/kg/day. Note that this model suggests a threshold at 0.005 mg/kg/day, which is slightly below the NOEL for the study at 0.007 mg/kg/day. This is because there is a measured decrease in iodine uptake (1.8%) even at the NOEL, although this decrease is not statistically significant. A comparison point for risk here is the NRC (2005) observation that a 75% decrease in iodide uptake would be required to initiate a potential health effect, although again, it must be noted that the percent decrease required in the sensitive subpopulation currently is unknown and is likely to be less than this value. As before, note that our assumption here is that the dose–response data from Greer et al. (2002) reflect the incremental decrease in iodide uptake per unit incremental increase in exposure to perchlorate through water alone, above and beyond the modifying effects of the background perchlorate exposures through other routes.

## Results

The Monte Carlo assessment was conducted for hypothetical MCLs of 1, 2, 5, 6, 10, 20, 24.5, and 50 μg/L (values of 6 and 24.5 were included to reflect potential limits by the California Environmental Protection Agency and the U.S. EPA DWEL, respectively). The analysis was conducted first using the national occurrence distribution to reflect nationwide conditions. In this analysis the actual distribution of perchlorate concentrations in CWSs is assumed (median of 0.03 μg/L and GSD of 13), with systems above the MCL mitigated to exactly the MCL (the nonexceeding systems remain at their current concentrations). From this, the distribution of water concentrations in the United States was established after the MCL is in place, and a value was selected at random. An intake rate per unit body mass for an individual in the sampled population (women of child-bearing age) then was selected at random from the distribution described previously (median of 0.0182 L/kg/day and a GSD of 1.8). The product of the perchlorate concentration in water and the intake rate of water per unit body weight then equals the ADRI for that sampled individual. The sampled ADRI was divided by the RfD (0.007 mg/kg/day) to produce an estimate of the HQ, then the ADRI was placed into the model in Figure 1 to produce an estimate of percentage reduction in iodine uptake. The Monte Carlo process was repeated for 10,000 individuals to generate intersubject variability distributions for these two risk metrics. The value of 10,000 was based on the goal of providing stability in the tails of the distribution.

Then we focused on intersubject variability of doses and risk metrics for people possibly exposed to water mitigated to exactly a potential MCL to reflect risk distributions only within those CWSs that currently have elevated perchlorate concentrations and might therefore be expected to reduce concentrations down to the MCL. The Monte Carlo process is the same as described previously (including the focus on the sensitive subpopulation), with the exception that all individuals are exposed at the same concentration of perchlorate in water, equal to the MCL. Both sets of results are described below.

### National occurrence results

The HQ results using the national occurrence analysis are summarized in Table 1. For example, at the 95th percentile of the sensitive subpopulation, the HQ value was 0.02 (i.e., dose was 2% of the RfD no risk threshold), even at an MCL of 50 μg/L. Note from this same table that the percent decrease in iodine uptake, using the model in Figure 1, is zero for all MCL values and percentiles examined because the ADRI was below the threshold in the model.

### Results for systems at the MCL

In this second set of calculations, all individuals in the sensitive subpopulation are assumed exposed at the concentration of a potential MCL. In other words, in this analysis, we examine risks to the highly exposed portion of the sensitive subpopulation after the potential MCL has been established and all CWSs are mitigated down to that MCL.

For the at-the-MCL analysis, some HQ values do exceed 1.0. As shown in Table 2, there were no HQ values > 1.0 at MCLs of ≤ 24.5 μg/L for the percentiles of the cumulative distribution functions examined. In systems with perchlorate concentrations of 50 μg/L, however, 28.6% of the sensitive subpopulation had an HQ value exceeding 1.0 (an HQ value of 1.0 was found at approximately the 71st percentile of the variability distribution for this population). At the 90th percentile, the HQ value at 50 μg/L exposure was 1.54, and at the 95th percentile the HQ value was 1.89. There was, however, no reduction in iodide uptake estimated from the model at any MCL because all intake rates were below the threshold for the model in Figure 1.

### Sensitivity analyses

The above-described analyses and results are based on several assumptions that can be altered. We conducted several alternate Monte Carlo simulations to reflect a mix of potential differences in selection of underlying data or in how those data are interpreted. The goal here was to determine an upper-bound estimate of the risks, and so more conservative assumptions were used than was the case in Tables 1 and 2. Specifically, in this new analysis, the amount of water consumed was increased to include total water intake (not just intake from CWSs), as obtained from the U.S. EPA *Exposure Factors Handbook* (U.S. EPA 1999). Using the national occurrence data for the concentration of perchlorate in the drinking water (i.e., assuming the non-CWS concentration was the same as that in the CWS for an individual), there is no appreciable difference between the base case results from Table 1 and the “upper-end” values calculated here. Results in Table 1 may therefore be assumed to represent upper-end risks when all water consumption, and not only drinking water, is considered in the exposure assessment.

However, for the at-the-MCL analysis results (as shown in Table 3, and equivalent to Table 2), for those women consuming water with perchlorate at the potential MCLs, there are some elevated HQ values compared with those in the base analysis depicted in Table 2. In particular, there are now HQ values > 1.0 at the 95th percentile even at 20 μg/L.

## Discussion and Conclusions

Perchlorate in drinking water is more widespread than originally anticipated, with perhaps 2% of sources showing detectable levels ≥ 4 μg/L. Combining the newly emerging risk and occurrence information, we have modeled the percentage of the sensitive subpopulation (pregnant women) that may face (or whose infants may face) a risk of adverse health effects due to perchlorate in U.S. drinking waters. The results indicate that for any population using a CWS with a perchlorate concentration of 50 μg/L (i.e., slightly more than twice the proposed U.S. EPA DWEL of 24.5 μg/L), there would be an appreciable percentage of pregnant women who face a risk of adverse effects in themselves or their fetuses because they would have an HQ value > 1.0. When perchlorate concentrations are 50 μg/L, between 28.6% (if only ingestion of drinking water is assumed) and 58.1% (if all water ingestion is assumed, with the non-CWS being similarly contaminated by perchlorate) of the sensitive subpopulation might face a dose exceeding the RfD. Values of the HQ > 1.0 at the 95th percentile of the intersubject variability distribution are predicted at 20 μg/L perchlorate if ingestion of all water, and not only drinking water, is included in the exposure assessment. The results suggest that few women in the sensitive subpopulation would face a significant perchlorate risk from drinking water at MCLs ≤ 24.5 μg/L if only drinking water is considered, but that the equivalent MCL would need to be slightly below 20 μg/L if all water ingestion were considered.

We caution the reader on the interpretation of these results. The present analysis falls within a framework of probabilistic risk assessment that differs in significant ways from traditional approaches to determining regulatory limits on exposure. In those traditional approaches, risks are estimated to maximally exposed individuals within sensitive subpopulations, and the concentration determined that produces an acceptable level of risk in those individuals. This level is independent of any consideration of the fraction of people in that subpopulation. The question being addressed traditionally is to what extent a proposed MCL will reduce the risk to an individual in this maximally exposed, sensitive subpopulation.

Probabilistic risk assessment as conducted here, however, examines the intersubject variability distribution of risks in this sub-population and asks what fraction of people in an exposed population have a risk (HQ or percentage decrease in iodide uptake) judged to be unacceptable. Such probabilistic distributions form the basis of cost–risk–benefit calculations, allowing society to determine how a given mode of risk reduction (e.g., controls on perchlorate exposures) compares against other modes of risk reduction. The goal then is to determine the total burden of disease in a population and to use this estimate of burden to determine whether the examined mode of risk reduction (here, control on perchlorate exposures) represents an effective way to allocate limited societal resources in improving the overall health of the public. We have not attempted here to draw any conclusions in that regard, but rather to present the probabilistic information on which such cost–risk–benefit assessments might be based.

## Figures and Tables

**Table 1:** HQ values for pregnant women (the sensitive subpopulation): base case analysis, using national occurrence data (i.e., existing distribution of perchlorate in water, with only supplies currently above the proposed MCL mitigated down to the proposed MCL).

MCL (μg/L)	Median^a^	90th percentile^b^	95th percentile^b^	Percent HQ < 1^c^	Percent decrease^d^
1	0.01	0.02	0.02	> 99	0
2	0.01	0.02	0.02	> 99	0
5	0.01	0.02	0.02	> 99	0
6	0.01	0.02	0.02	> 99	0
10	0.01	0.02	0.02	> 99	0
20	0.01	0.02	0.02	> 99	0
24.5	0.01	0.02	0.02	> 99	0
50	0.01	0.02	0.02	> 99	0

a The median for the variability distribution.

b The 90th and 95th percentiles of the variability distribution.

c The percentage of the population with an HQ value < 1.

d The percent decrease in iodide uptake for individuals at the 95th percentile. The percent decrease is predicted using the equation in the text; a value of 0% indicates the modeled threshold of 0.005 mg/kg/day has not been exceeded.

**Table 2:** HQ values for pregnant women (the sensitive subpopulation): base case analysis, for persons using CWSs at the MCL concentration (i.e., considering only supplies currently above a potential MCL, which are mitigated down to the potential MCL).

MCL (μg/L)	Median^a^	90th percentile^b^	95th percentile^b^	Percent HQ < 1^c^	Percent decrease^d^
1	0.01	0.03	0.04	> 99	0
2	0.03	0.06	0.08	> 99	0
5	0.07	0.15	0.19	> 99	0
6	0.08	0.19	0.22	> 99	0
10	0.14	0.30	0.38	> 99	0
20	0.29	0.62	0.76	> 99	0
24.5	0.33	0.70	0.90	> 99	0
50	0.73	1.54	1.89	71.4	0

a The median for the variability distribution.

b The 90th and 95th percentiles of the variability distribution.

c The percentage of the population with an HQ value < 1.

d The percent decrease in iodide uptake for individuals at the 95th percentile. The percent decrease is predicted using the equation in the text; a value of 0% indicates the threshold of 0.005 mg/kg/day has not been exceeded.

**Table 3:** HQ values for pregnant women (the sensitive subpopulation): sensitivity analysis, high-end exposure scenarios for persons consuming all water, and not only drinking water, at the MCL.

MCL (μg/L)	Median^a^	90th percentile^b^	95th percentile^b^	Percent HQ < 1^c^	Percent decrease^d^
1	0.04	0.07	0.08	> 99	0
2	0.06	0.11	0.13	> 99	0
5	0.13	0.25	0.31	> 99	0
6	0.15	0.28	0.36	> 99	0
10	0.24	0.48	0.60	> 99	0
20	0.45	0.95	1.16	91.2	0
24.5	0.50	1.10	1.35	88.3	0
50	1.10	2.37	2.90	41.9	0

a The median for the variability distribution.

b The 90th and 95th percentiles of the variability distribution.

c The percentage of the population with an HQ value < 1.

d The percent decrease in iodide uptake for individuals at the 95th percentile. The percent decrease is predicted using the equation in the text; a value of 0% indicates the threshold of 0.005 mg/kg-day has not been exceeded.

## Appendix. Mode of action for perchlorate

There is broad scientific agreement that the mode of action for perchlorate is as follows:

- Perchlorate binds to, and blocks, receptors for the movement of iodine from the bloodstream into the thyroid. This will reduce the movement of iodine into the thyroid.
- The thyroid responds to this reduction either by producing less triiodothyronine (T_3_) and thyroxine (T_4_) or by drawing on the pool of iodine stored in the thyroid.
- Perchlorate can therefore reduce the production of T_3_ and T_4_ initially, although there are feedback mechanisms that can bring these levels in the circulating blood back to normal ranges over time.
- The effect on T_4_ is not significant because that molecule is an intermediary, and so the focus should be on changes in T_3_.
- For some fraction of the population that has very little iodine stored in the thyroid, there may be reduced ability to compensate for the reduction of iodine crossing from the blood-stream to the thyroid. This may reduce T_3_ levels in the circulating blood, leading eventually to increased production of thyroid-stimulating hormone (TSH) to try to correct the imbalance.
- If the imbalance cannot be corrected, there could be changes in metabolic function at any age of exposure, or abnormal fetal and child growth and development.

The NRC committee (NRC 2005) disagreed as to where the U.S. EPA should draw the line between adverse and nonadverse effects. The agency considered changes in T_3_ and TSH levels to be adverse in and of themselves, or at least indications of, or bio-markers of, adverse effects. The NRC committee did not consider changes in T_3_ and/or TSH adverse in and of themselves. Instead, the NRC committee claimed that changes in these levels must first produce thyroid hypertrophy or hyperplasia, followed by hypothyroidism, which then will produce the final metabolic and growth/developmental effects mentioned above. The NRC committee considered the first effect that is adverse to be hypothyroidism rather than either the preceding thyroid hypertrophy/hyperplasia or the changes in T_3_ and/or TSH.
